# Supplementary material for: Organic matter loading by hippopotami causes subsidy overload resulting in downstream hypoxia and fish kills
Source: Nat Commun. 2018 May 16;9:1951. doi: 10.1038/s41467-018-04391-6 (PMC5956076; doi:10.1038/s41467-018-04391-6)
Supplement: Supplementary file 1 — Supplementary Information [file 41467_2018_4391_MOESM1_ESM.pdf]

Supplementary Materials

Organic matter loading by hippopotami causes subsidy overload resulting in downstream hypoxia and fish kills

Dutton et al.

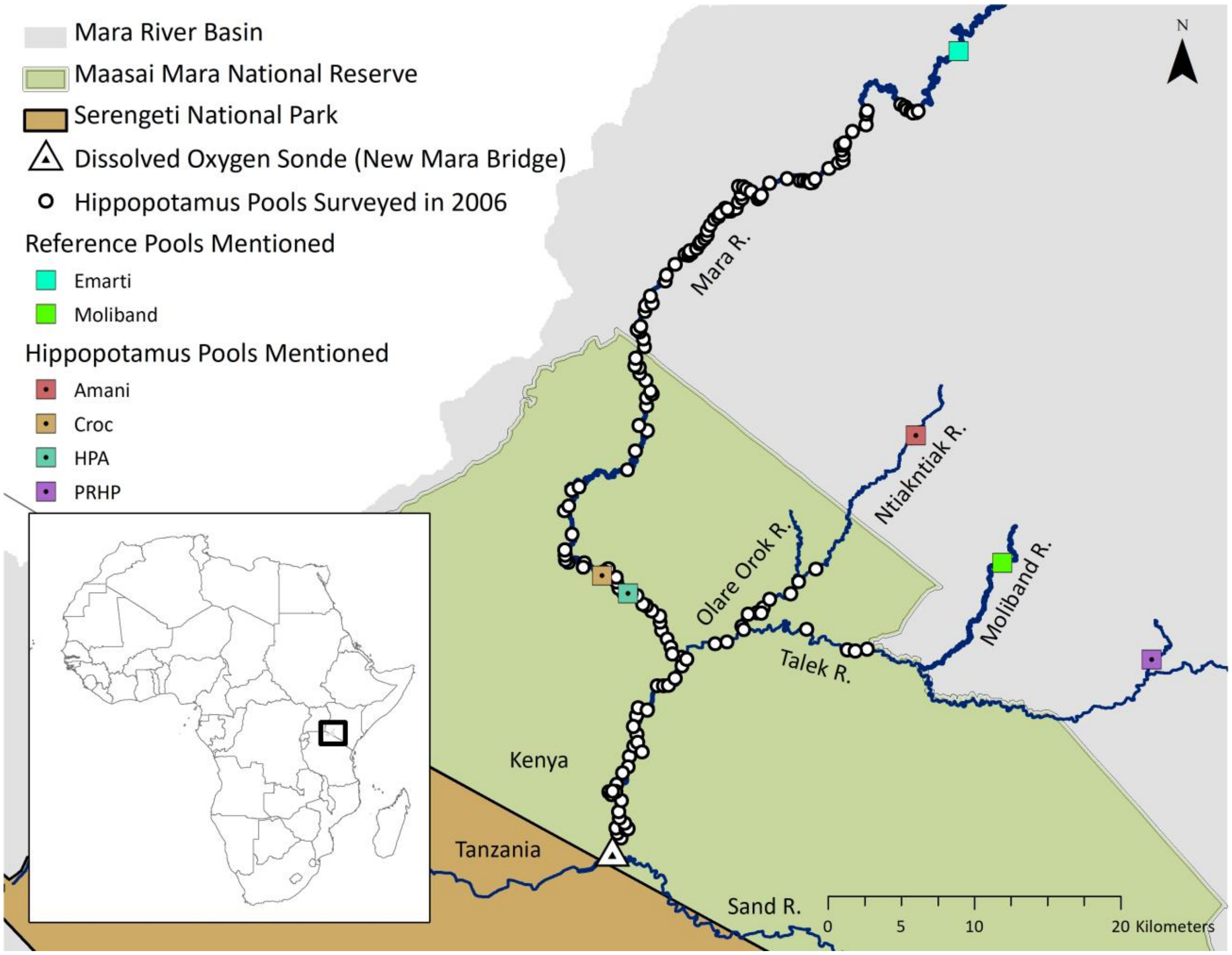

Supplementary Figure 1.

Map of the study area showing the relationship of hippo pools surveyed in 2006[1], the dissolved oxygen sonde at New Mara Bridge and the hippo pools and reference pools used as part of this study. Map was created in ArcMap 10.2.2 (ESRI, Redlands, CA, USA).

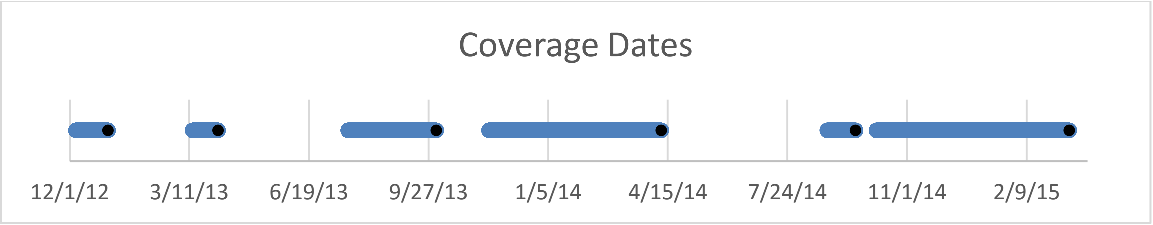

Supplementary Figure 2.
Coverage dates for the dissolved oxygen loggers.

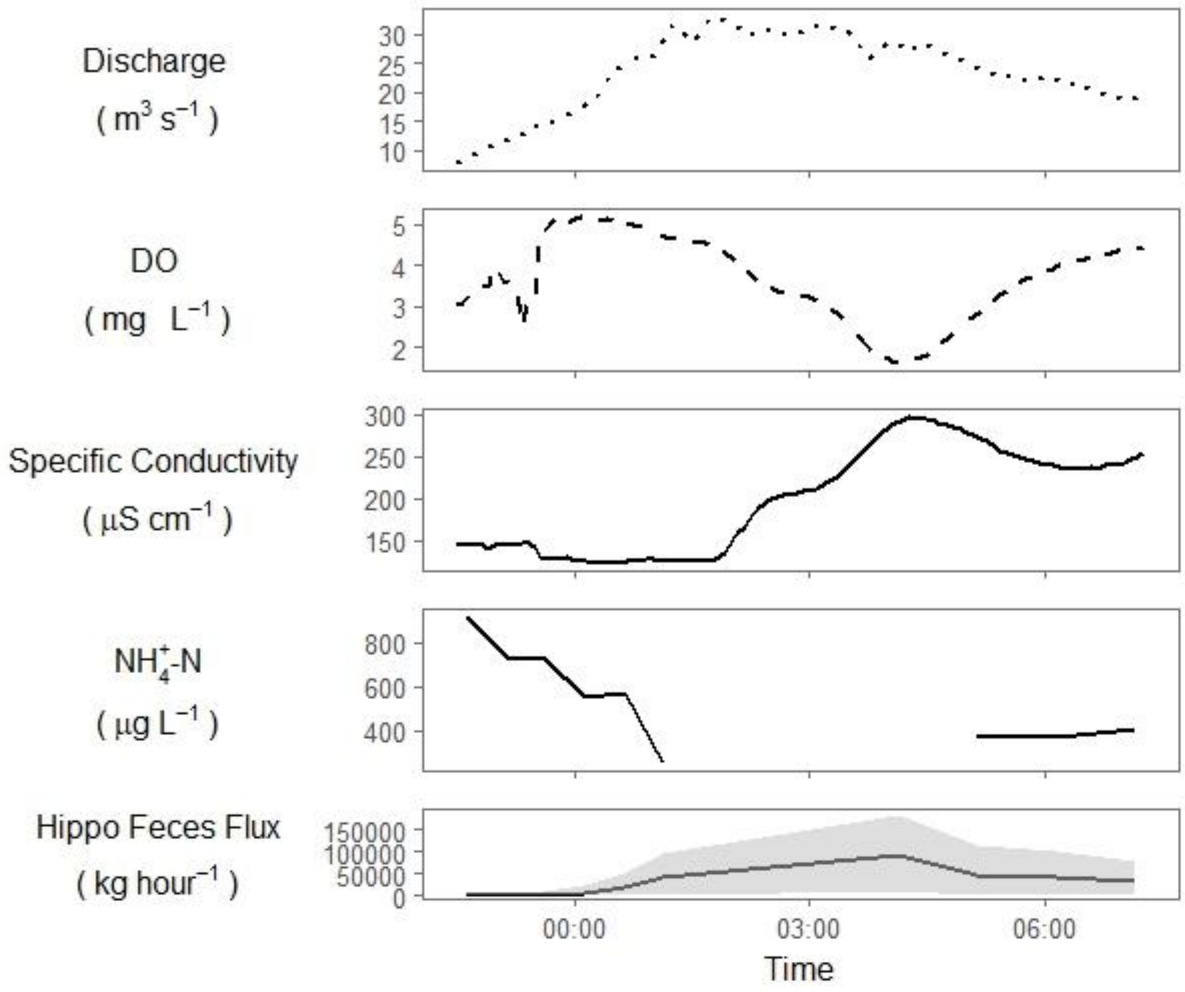

Supplementary Figure 3.

Discharge, dissolved oxygen (DO) concentrations, specific conductivity, NH_4^+-N and the flux of hippopotamus feces (estimated by sediment fingerprinting) for the flood on October 27-28 2012. Bayesian 95% credibility intervals for the flux of hippopotamus feces is in grey. A gap in the line for NH_4^+-N indicates missing data.

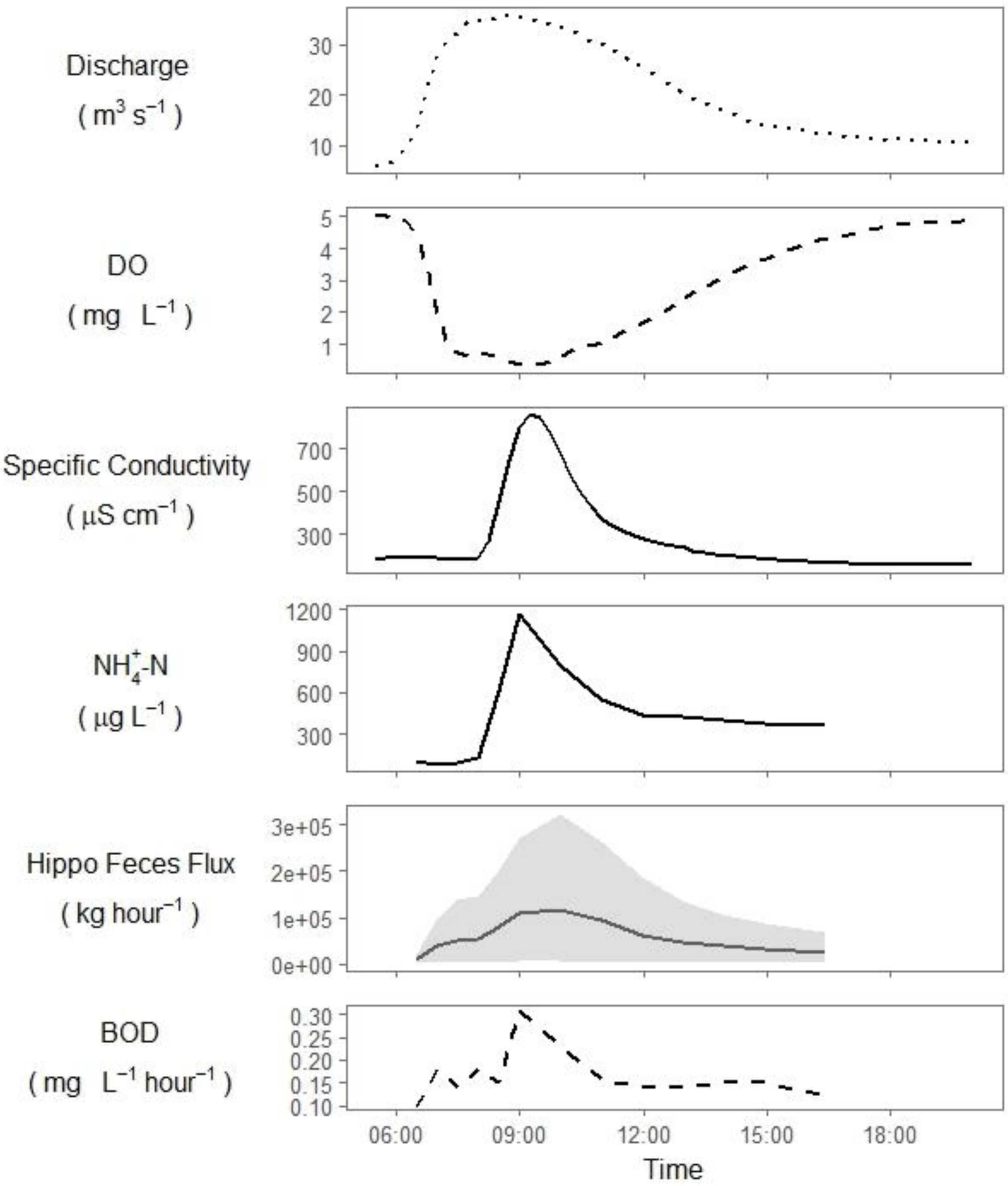

Supplementary Figure 4.

Discharge, dissolved oxygen (DO) concentrations, specific conductivity, NH_4^+-N, hippopotamus feces fluxes (estimated by sediment fingerprinting), and biochemical oxygen demand (BOD) for the flood on December 10, 2013. Bayesian 95% credibility intervals for the flux of hippopotamus feces is in grey.

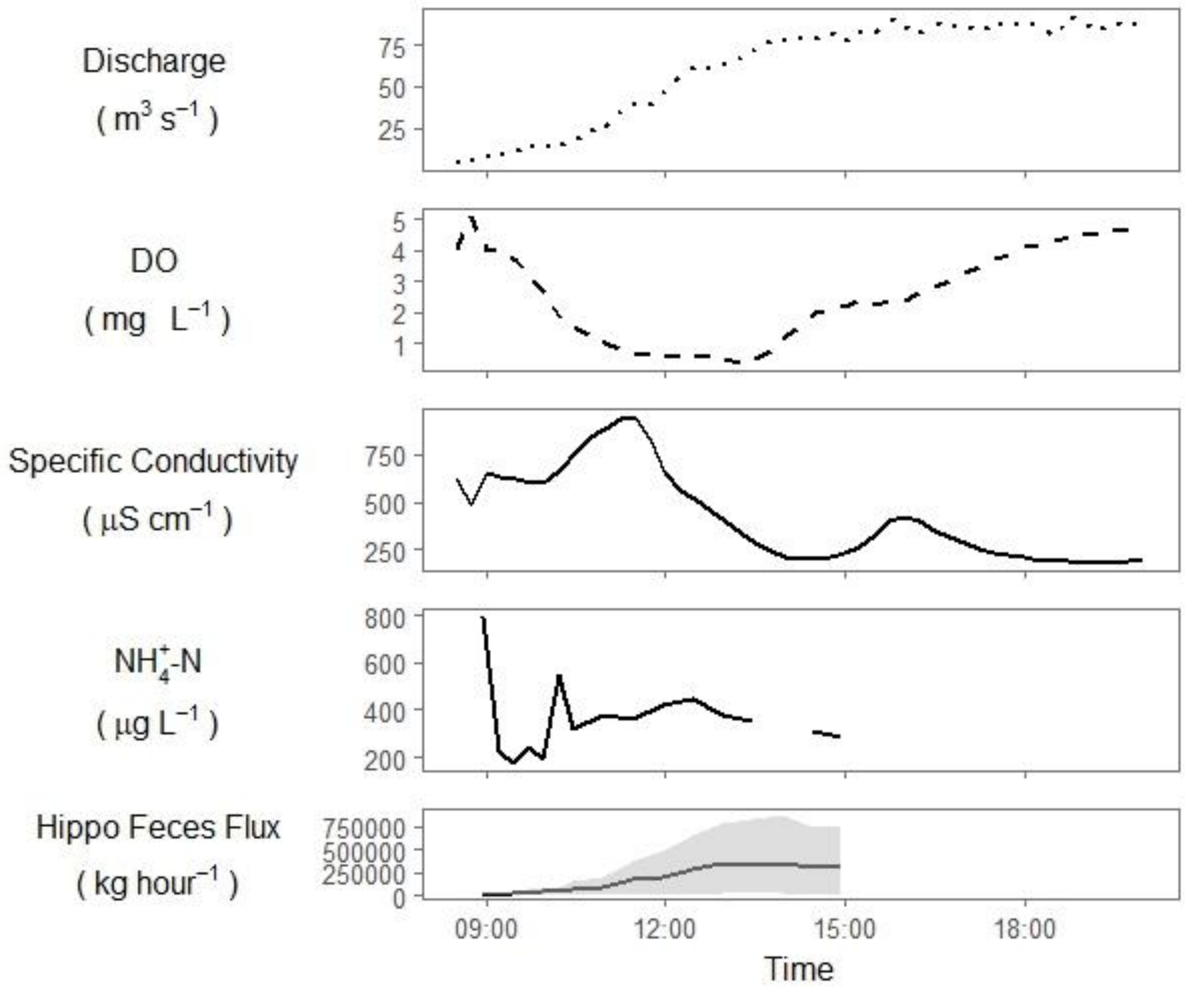

Supplementary Figure 5.

Discharge, dissolved oxygen (DO) concentrations, specific conductivity, NH_4^+-N, and hippopotamus feces fluxes (estimated by sediment fingerprinting) for the flood on March 14, 2014. Bayesian 95% credibility intervals for the flux of hippopotamus feces is in grey. A gap in the line for NH_4^+-N indicates missing data.

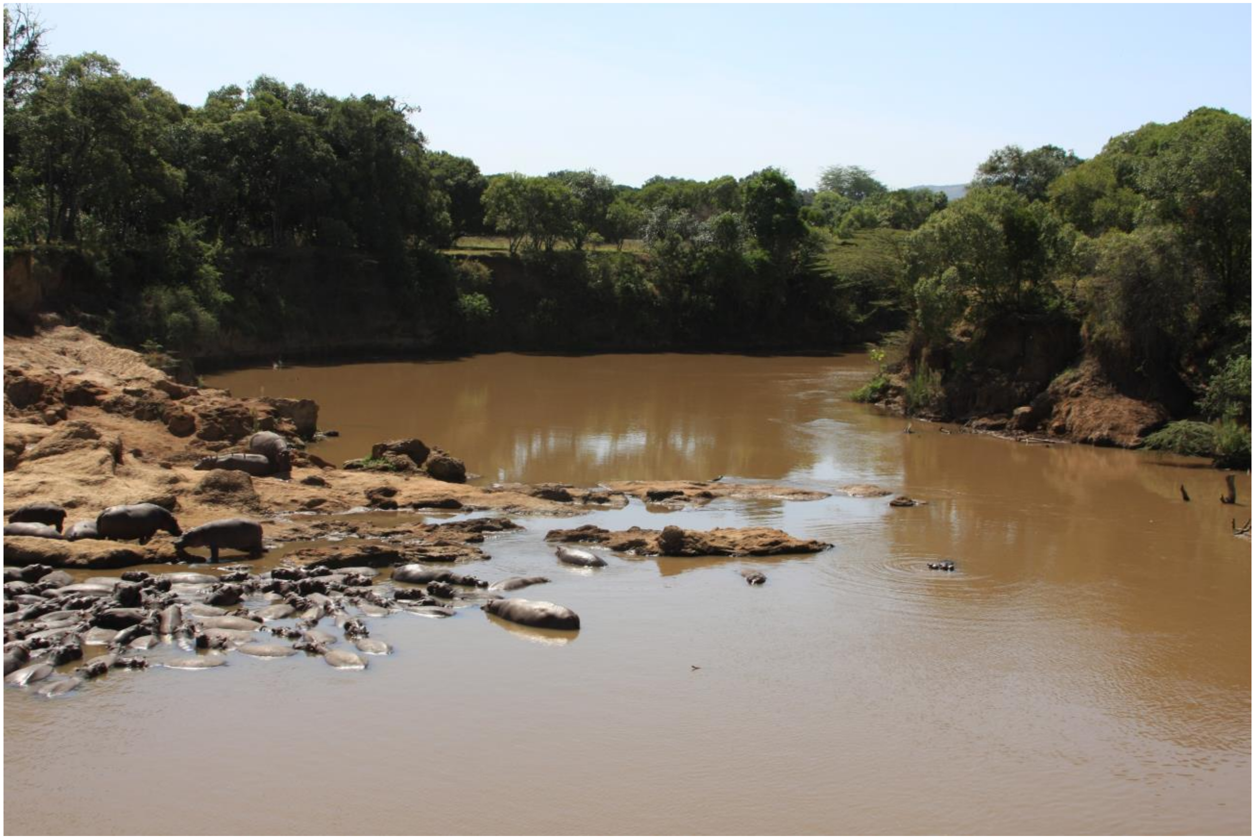

Supplementary Figure 6.
Picture of the deeply incised river channel upstream of the Ngerende hippo pool during baseflow (image credit: Christopher Dutton). Picture taken on 07 August 2008 at -1.094, 35.199. Channel is approximately 40 meters wide. The bank is approximately 3-6 meters higher than the river.

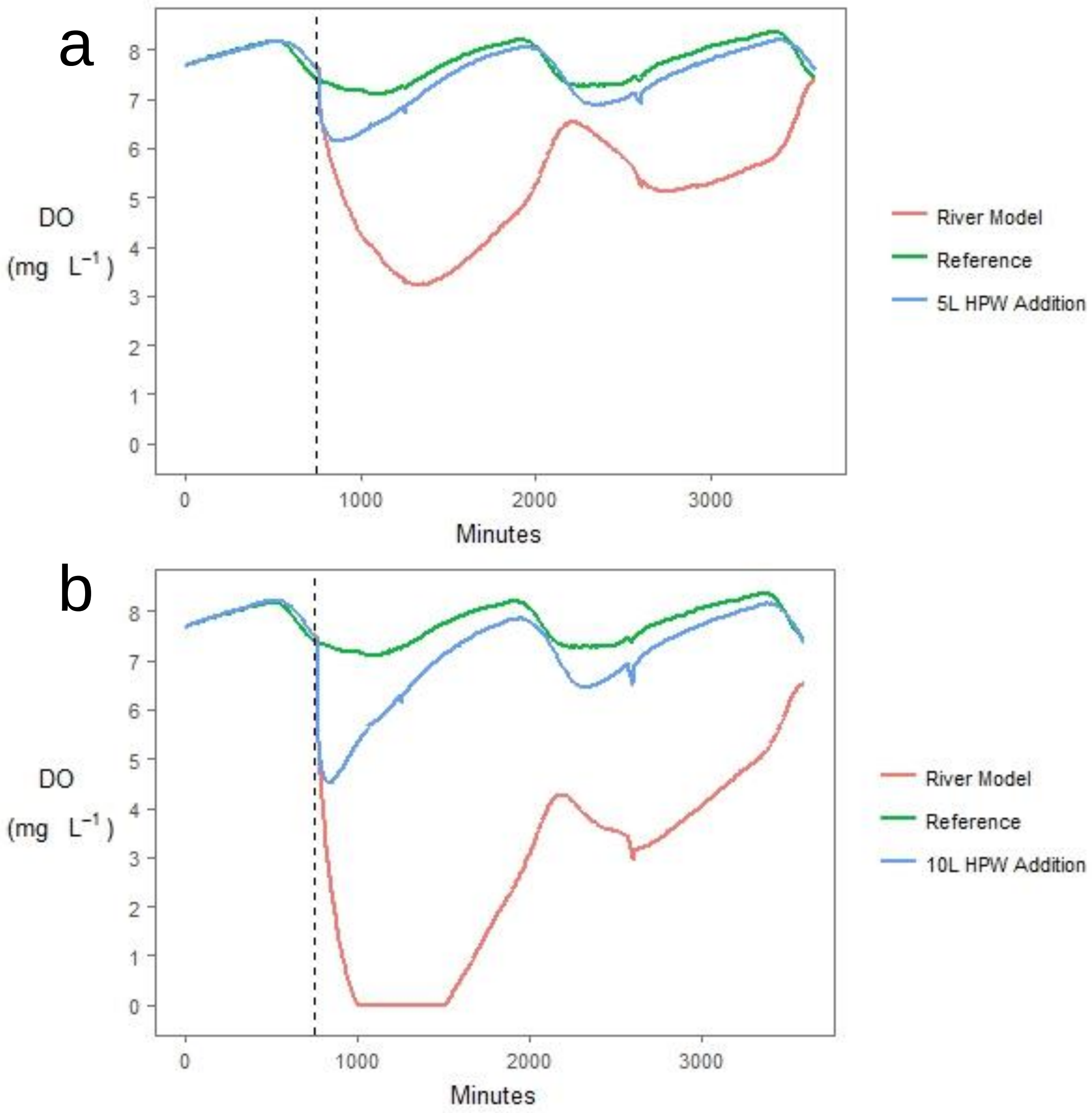

Supplementary Figure 7. Model results extrapolating the DO observations in the experimental stream channel to a deeper river channel similar to the Mara River. (a) Results based on addition of 5 L of hippo pool water (HPW) to 55 L of upstream river water; (b) Results based on addition of 10 L of HPW to 50 L of upstream river water. The HPW was added at 745 minutes (dashed line) and took a few minutes to fully mix. The diel periodicity is explained by water temperature, which varied between 14.2-24.4 °C during the experiment; the minimum daily DO concentrations in the experiment coincided with maximum water temperatures.

Supplementary Table 1.

Details for the 9 fish kills documented between 2009 and 2015.

Date	Observed Location	River	Reported by	Species
24-Feb-2009	NMB	Mara	Mara Conservancy Rangers	multiple species[1]
16-Nov-2009	NMB	Mara	Authors	multiple species[1]
3-Feb-2011	OMB to NMB	Mara	Mara Conservancy Rangers	*Mormyrus kannume*, *Barbus* sp., *Clarias gariepinus*
15-Jun-2011	NMB	Mara	Authors	primarily *Mormyrus kannume*
10-Sep-2012	NMB	Mara	Authors	multiple species[1]
10-Dec-2013	NMB	Mara	Authors	*Labeo victorianus, Labeobarbus altianalis, Barbus* sp., *Mormyrus kannume*
25-Mar-2014	Serena pump house	Mara	Rekero Lodge Guides	multiple species[1]
17-Feb-2015	NMB	Talek, Mara	Rekero Lodge Guides	multiple species[1]
28-Mar-2015	NMB	Mara	Authors	multiple species[1]

NMB = New Mara Bridge; OMB = Old Mara Bridge.

[1] Multiple fish species were observed but inaccessible for accurate identification.

Supplementary Table 2.

Remote controlled boat survey of pools.

Pool	Date	Discharge (m^3 s^{-1})	# Hippos	Depth (cm)	Average Conductivity (µS cm^{-1})	
					Surface	Benthos
Emarti	2-Feb-2015	2	0	111	347	1776
Moliband	23-Jan-2015	<0.1	0	67	300	382
Amani	4-Feb-2015	<0.1	13	122	811	2788
Croc	30-Jan-2015	2	20	100	424	1604
HPA	30-Jan-2015	2	9	106	689	1717

Supplementary Table 3.
Hydrochemical variables measured in the HPW used in the experiments.

	Hippopotamus Pool Water (HPW)	
Origin	Amani	PRHP
Use	Bottle and Stream Experiments	Pool Flushing Experiment
NH_4^+-N (µg L^{-1})	10632.3	25217.6
NO_3^--N (µg L^{-1})	41.7	29.5
SRP (µg L^{-1})	176.1	2766.6
TN (mg L^{-1})	22.3	28.0
TP (µg L^{-1})	2000.9	3233.8
DOC (mg L^{-1})	27.3	73.0
H_2S (µg L^{-1})	12.0	1196.0
Fe(II) (mg L^{-1})	0.4	0.2
CO_2 (µmol L^{-1})	1051.1	1621.3
CH_4 (µmol L^{-1})	89.1	220.7
N_2O (µmol L^{-1})	0.0	0.0
F^- (mg L^{-1})	1.9	2.2
Cl^- (mg L^{-1})	31.9	44.4
Br^- (mg L^{-1})	0.2	0.3
SO_4^{2-} (mg L^{-1})	23.7	47.8
Na^+ (mg L^{-1})	61.9	83.3
K^+ (mg L^{-1})	51.4	63.3
Mg^{2+} (mg L^{-1})	8.7	9.4
Ca^{2+} (mg L^{-1})	47.1	51.1
BOD5 (mg L^{-1})	420.0	993.8

SRP = soluble reactive P; TN = total nitrogen; TP = total phosphorus; DOC = dissolved organic carbon; SpCond = specific conductance, corrected to 25 °C; BOD5 = biological oxygen demand over 5 days.

Supplementary Table 4.

Discharge and hydrochemical variables measured during the flood pulse on 27-28 October 2012 at New Mara Bridge. Data from the sonde is presented at the same resolution as data from the water samples captured by the ISCO water sampler.

Time	Q ($m^3 s^{-1}$)	DO ($mg L^{-1}$)	TSS ($mg L^{-1}$)	TN ($mg L^{-1}$)	TP ($\mu g L^{-1}$)	DOC ($mg L^{-1}$)	NH_4^+-N ($\mu g L^{-1}$)	NO_3^--N ($\mu g L^{-1}$)	SRP ($\mu g L^{-1}$)	SpCond ($\mu S cm^{-1}$)
22:38	7.9	3.26	283	3.0	257	2.72	913	710	49	144.8
23:08	12.5	3.59	215	2.6	239	2.59	737	883	34	144.7
23:38	14.6	5.01	269	2.5	224	3.45	722	1010	55	127.9
0:08	19.0	5.18	398	2.6	256	2.76	553	975	33	125
0:38	25.8	5.03	653	3.0	324	2.65	571	993	35	125.5
1:08	31.7	4.69	999	3.1	413	3.01	255	907	17	125.2
4:08	27.1	1.62	3002	9.2	1325	7.87		138	19	292.3
5:08	26.5	2.89	2915	8.4	1270	5.05	385	253	29	270.9
6:08	23.5	3.99	2881	8.2	1472	4.93	372	294	9	238.4
7:08	19.3	4.41	2541	7.1	1045	4.92	408	345	10	249.3

Q = discharge; DO = dissolved oxygen; TSS = total suspended sediments; TN = total nitrogen; TP = total phosphorus; SRP = soluble reactive P; DOC = dissolved organic carbon; SpCond = specific conductance, corrected to 25 °C.

Supplementary Table 5.

Discharge and hydrochemical variables measured during the flood pulse on December 10, 2013 at New Mara Bridge. Data from the sonde is presented at the same resolution as data from the water samples captured by the ISCO water sampler.

Time	Q (m^3 s^{-1})	DO (mg L^{-1})	TSS (mg L^{-1})	TN (mg L^{-1})	TP (µg L^{-1})	NH_4^+-N (µg L^{-1})	NO_3^--N (µg L^{-1})	DOC (mg L^{-1})	SpCond (µS cm^{-1})	pH (units)
6:29	13	4.39	1014	4.5	650	105	1088	4.1	187	7.83
6:59	28	1.96	2615	8.6	1231	83	731	3.5	183	7.8
7:29	32	0.72	3629	9.7	1365	97	732	4.1	181	7.09
7:59	35	0.67	3591	10.0	1409	126	714	4.3	184	7.1
8:29	35	0.58	4253	12.4	1661	610	357	8.7	451	7.12
8:59	35	0.38	5784	17.9	2531	1170	304	13.6	800	7.8
9:59	33	0.64	7786	16.7	2473	799	326	9.1	678	8.14
10:59	30	1.08	6839	13.5	1982	553	468	6.3	377	8.19
11:59	26	1.68	5993	12.4	1772	445	525	5.4	280	8.09
12:59	20	2.44	6109	11.9	1699	428	510	5.1	232	7.96
13:59	17	3.15	5256	11.3	1605	400	531	5.4	199	7.46
14:59	14	3.7	5140	10.7	1654	379	569	5.5	181	7.41
16:26	12	4.29	4265	10.1	1377	365	563	4.6	164	7.82

Q = discharge; DO = dissolved oxygen; TSS = total suspended sediments; TN = total nitrogen; TP = total phosphorus; SRP = soluble reactive P; DOC = dissolved organic carbon; SpCond = specific conductance, corrected to 25 °C.

Supplementary Table 6.

Discharge and hydrochemical variables measured during the flood pulse on March 14, 2014 at New Mara Bridge. Data from the sonde is presented at the same resolution as data from the water samples captured by the ISCO water sampler.

Time	Q (m^3 s^{-1})	DO (mg L^{-1})	TSS (mg L^{-1})	TN (mg L^{-1})	TP (µg L^{-1})	NH_4-N (µg L^{-1})	NO_3-N (µg L^{-1})	SRP (µg L^{-1})	DOC (mg L^{-1})	SpCond (µS cm^{-1})
8:57	8	4	1528	4.6	630	793	415	3	9.2	659
9:12	10	4.03	1012	4.7	657	226	200	3	8.5	638
9:27	11	3.68	1637	5.7	837	176	360	5	7.5	619
9:42	14	3.11	2351	8.5	1213	239	341	3	6.6	603
9:57	14	2.57	2841	9.0	1265	188	336	3	7.5	608
10:12	15	1.79	3112	9.5	1367	546	71	2	7.6	674
10:27	19	1.47	3705	10.0	1553	319	173	3	8.4	773
10:57	26	0.98	4448	11.0	1649	380	123	4	8.7	883
11:27	39	0.69	5194	11.8	1767	359	284	3	8.3	950
11:57	48	0.53	7790	11.0	1777	422	36	5	6.2	657
12:27	61	0.64	7524	12.2	1926	446	271	4	6.1	512
12:57	63	0.51	7436	12.9	1957	381	238	2	5.8	396
13:27	73	0.48	8291	12.6	1977	355	373	3	5.2	278
13:57	77	1.18	7983						4.1	213
14:27	78	1.95	6819	10.6	1629	311	338	5	3.2	197
14:57	78	2.15	6560	9.1	1444	282	257	10	4.6	229

Q = discharge; DO = dissolved oxygen; TSS = total suspended sediments; TN = total nitrogen; TP = total phosphorus; SRP = soluble reactive P; DOC = dissolved organic carbon; SpCond = specific conductance, corrected to 25 °C.

Supplementary Table 7.

Hydrochemical variables measured in the three treatments at the beginning and end of the bottle experiment (means with standard deviation in italics).

Treatment	HPW		Hippopotamus Feces		Control	
Minutes	30	1680	30	1680	30	1680
DO (mg L^{-1})	5.3<br>*0.1*	0.2<br>*0*	6.5<br>*0.2*	1.9<br>*0.4*	6.8<br>*0.2*	6.4<br>*0.1*
H_2S (µg L^{-1})	0<br>*0*	0.1<br>*0*	3.6<br>*0.4*	0<br>*0*	2.3<br>*0.7*	2.5<br>*0*
NH_4^+-N (µg L^{-1})	2142.8<br>*44.9*	2282.7<br>*101.8*	133.7<br>*84.5*	456.4<br>*315.6*	18.5<br>*29.5*	13<br>*13.2*
SRP (µg L^{-1})	10.2<br>*1.6*	180<br>*14.6*	147.3<br>*24.7*	602.2<br>*197.6*	14.2<br>*2.4*	7.4<br>*5.5*
NO_3^--N (µg L^{-1})	9.3<br>*1.6*	4.1<br>*0.4*	15.3<br>*18.1*	15.1<br>*4.7*	0<br>*0*	0<br>*0*
CO_2 (µmol L^{-1})	45.6<br>*4.2*	53.4<br>*1.3*	31<br>*8.6*	31.3<br>*5*	30<br>*3.2*	24<br>*2.2*
CH_4 (µmol L^{-1})	14.2<br>*10*	7.7<br>*0.9*	0.3<br>*1.6*	0<br>*0*	0<br>*0*	0<br>*0*

Supplementary Table 8.

Location, area and approximate volume of 14 hippo pools.

Hippo Pool	Location (Latitude, Longitude)	River	Area (m^2)	Approximate Volume (m^3)
PRHP	-1.4338861, 35.3499556	Upper Talek	960	960
Amani	-1.29539, 35.205	Ntiakntiak	1400	1400
Olare Orok Confluence	-1.41587, 35.09783	Talek	4200	4200
Smelly	-1.40463, 35.10347	Olare Orok	1610	1610
Double Cross	-1.38183, 35.13717	Ntiakntiak	1560	1560
Nbig	-1.42587, 35.35912	Upper Talek	2250	2250
Ngerende	-1.09401, 35.19948	Mara	6000	6000
Conservancy	-1.36837, 34.99061	Mara	4250	4250
Croc	-1.38198, 35.01229	Mara	6460	6460
Falls	-1.39032, 35.02396	Mara	5200	5200
HPA	-1.3931, 35.02828	Mara	3900	3900
Serena Picnic	-1.53826, 35.02628	Mara	4200	4200
Big	-1.54656, 35.02188	Mara	7918	7918
Rocky Crossing	-1.41828, 35.35728	Upper Talek	560	560

Supplementary Table 9.

Kruskal-Wallis test results from the sediment fingerprinting.

Element	Chi-Squared	Degrees of Freedom	p-value
P	32.52	3	< 0.001
Na	29.7	3	< 0.001
V	25.758	3	< 0.001
Ti	18.673	3	< 0.001
Ga	18.481	3	< 0.001
Fe	17.955	3	< 0.001
Pb	16.538	3	0.001
Ni	15.992	3	0.001
U	15.046	3	0.002
K	14.288	3	0.003
Sb	14.181	3	0.003
Cr	10.857	3	0.013
Zn	10.446	3	0.015
Ba	10.43	3	0.015
Mo	9.5327	3	0.023
Cu	7.4212	3	0.060

Supplementary Table 10.

Step-wise discriminant function analysis results from the sediment fingerprinting.

Step	Formula	Cumulative Error %
1	U	37%
2	U + Na	33%
3	U + Na + Ga	28%
4	U + Na + Ga + K	15%
5	U + Na + Ga + K + Pb	11%
6	U + Na + Ga + K + Pb + P	8%
7	U + Na + Ga + K + Pb + P + V	8%
8	U + Na + Ga + K + Pb + P + V + Zn	9%
9	U + Na + Ga + K + Pb + P + V + Zn + Ba	9%
10	U + Na + Ga + K + Pb + P + V + Zn + Ba + Mo	9%
11	U + Na + Ga + K + Pb + P + V + Zn + Ba + Mo + Fe	8%
12	U + Na + Ga + K + Pb + P + V + Zn + Ba + Mo + Fe + Ni	8%
13	U + Na + Ga + K + Pb + P + V + Zn + Ba + Mo + Fe + Ni + Cr	8%
14	U + Na + Ga + K + Pb + P + V + Zn + Ba + Mo + Fe + Ni + Cr + Ti	8%
16	U + Na + Ga + K + Pb + P + V + Zn + Ba + Mo + Fe + Ni + Cr + Ti + Sb	8%

Supplementary Table 11.

Confusion matrix for all potential sediment sources in the sediment fingerprinting mixing model.

	Hippo	Middle Mara	Talek	Upper Mara	Accuracy
Hippo	6	0	0	2	87.5%
Middle Mara	0	38	1	1	90.0%
Talek	1	0	8	4	61.5%
Upper Mara	0	2	1	15	77.8%

Supplementary Note 1.

Further consideration of nutrient patterns during flushing flows.

The changes in nutrient concentrations measured during the three flushing flows with automated samplers varied across flow events. Notably, NO_3^- and NH_4^+ declined with decreasing dissolved oxygen (DO) concentration during the October 2012 and March 2014 flushing flows. During the December 2013 flushing flow, in which a fish kill occurred, NH_4^+ rose and peaked during the DO decrease.

We would not expect the peak in NH_4^+ concentration to necessarily be in sync with a decrease in DO because nitrification would quickly consume excess NH_4^+ with even modest oxygenation, and even anoxic river water still receives oxygen influx from air-water gas exchange. Variation in the hydrological origin and magnitude of each flushing flow would also introduce variation in the DO and nutrient responses[2] (Fig 1b).

Additionally, soluble reactive P (SRP) concentrations were very low and did not show a consistent trend across all three flushing flows, yet we have measured very high concentrations of SRP in pools with hippopotami (Supplementary Table 3). One possibility is that iron oxyhydroxides precipitate upon mixing of anoxic pool water with inflowing river water, and the SRP is removed from solution by sorption, although we lack the measurements necessary to assess that hypothesis[3,4]. Another possibility is that SRP may sorb to clay sediments given the very high sediment loads experienced during the flushing flows (peak TSS concentrations reached as high as 8000 mg L^{-1} during the March 2014 flushing flow).

Supplementary Note 2.

Modeling downstream oxygen depletion

Diffusive gas exchange between the water and atmosphere was estimated from dissolved gas concentrations using the stagnant film model[5]:

$$F_{areal} = D\left[\frac{(Cobs - Ceq)}{z}\right] \quad \text{(S1)}$$

where F_{areal} = flux in nmol cm^{-2} s^{-1}; D = coefficient of molecular diffusion for the gas in cm^2 s^{-1}; C_{obs} = observed dissolved gas concentration in μM (=nmol cm^{-3}); C_{eq} = gas concentration (μM) in equilibrium with the atmosphere; and z = thickness of the hypothetical boundary layer (cm). Equilibrium DO concentrations (from Henry's Law) and diffusion coefficients were estimated for the measured water temperatures using the empirical relationship in Benson and Krause[6] and the Stokes-Einstein equation, respectively. A reasonable boundary layer thickness (z) for flowing water is 50 μm = 0.0050 cm. Water column depths: 15 cm in artificial channels, 100 cm in model river.

For time intervals preceding each DO measurement, we estimated the steady-state areal reaeration rate (F_{areal}) using the mean of the two temperature and DO measurements (i.e., before and after the interval) in the above equation.

The net DO concentration change in the water (Δ DO_{vol}, μM s^{-1}) was estimated from the change in DO concentration (μM) during the interval divided by interval time t in seconds (s):

$$\Delta DO_{vol} = \frac{C_t - C_0}{t} \quad \text{(S2)}$$

where C_t and C_0 are corrected for the reference concentrations ($C_{obs\ reference}$) at each time step.

The Δ DO_{vol} was converted to an areal rate (Δ DO_{areal}, nmol cm^{-2} s^{-1}) as follows, assuming a water depth of 15 cm in the artificial channels:

$$\Delta DO_{areal} = \Delta DO_{vol} * 10^3 \frac{nmol}{\mu mol} * 0.015 \frac{L}{cm^2} \quad \text{(S3)}$$

The gross DO consumption rate on an areal basis (R_{areal}, nmol cm^{-2} s^{-1}) was considered equal to the Δ DO_{areal} plus the reaeration rate (both have negative signs when DO is decreasing; effectively we are adding the DO coming in via reaeration):

$$R_{areal} = \Delta DO_{areal} - F_{areal} \quad \text{(S4)}$$

This gross DO consumption rate was expressed on a volumetric basis (R_{vol}, μM s^{-1}) as follows:

$$R_{vol} = R_{areal} * 10^{-3}\,\frac{\mu mol}{nmol} * \frac{1\ cm^2}{0.015\ L} \quad \text{(S5)}$$

We then applied this rate to the river. In the river model we use R_{vol} determined above for each time interval.

We predicted the river DO concentration (C_{pred}) as follows:

$$R_{areal\ river} = R_{vol} * 10^{3}\,\frac{nmol}{\mu mol} * \frac{0.1\ L}{cm^2} \quad \text{(S6)}$$

$F_{arealriver}$ was determined using the above equation and the water temperatures and DO concentration at the start of each interval (note that this refers to the C_{pred} below; we cannot use the mean of before and after each interval because that becomes a circular calculation).

$$\Delta DO_{areal\ river} = R_{areal\ river} - F_{areal\ river} \quad \text{(S7)}$$

$$\Delta DO_{vol\ river} = \frac{\Delta DO_{areal\ river}}{10^{3}\frac{nmol}{\mu mol} * \frac{0.1\ L}{cm^2}} \quad \text{(S8)}$$

$$C_{pred} = C_{t-1} + (\Delta DO_{vol\ river} * t) \quad \text{(S9)}$$

Once the C_{pred} reached negative values we considered the water to be anoxic.

Supplementary Note 3.

Sediment fingerprinting statistical method

Elemental composition data from suspended sediments were used as “downstream” samples in a sediment fingerprinting method along with previously collected source data from throughout the catchment[7]. Additional source samples for Upper Mara, Talek and Hippo were added to the original source signatures to represent a larger range of variability in those three source types (Upper Mara n=18, Talek n=12, Hippo n=8).

To identify the contribution of hippopotamus feces from the pools to downstream suspended sediments during flushing flows, the Kruskal-Wallis H test was first used to identify the elements that showed significant differences between the potential sources[8]. Out of the 16 elements assessed, copper was eliminated due to a p-value greater than 0.05 (Supplementary Table 9). A step-wise Discriminant Function Analysis (DFA) based on the minimization of Wilk’s lambda was then used to determine which combination of elements provided the best discrimination ability for the least number of elements[8-10]. The combination of uranium, sodium, gallium, potassium, lead and phosphorus provide the best discriminatory ability between the potential sources (Supplementary Table 10, cumulative error of 0.08%).

A jackknifed Discriminant Function Analysis (jDFA) was then utilized to provide an assessment of the discriminatory power of the tracers through a cross-validation procedure[9]. The jDFA runs multiple DFAs, leaving a different source out each time and attempting to guess the appropriate source classification for that sample. The jDFA found this combination of elements was able to classify the appropriate sample 86% of the time. Talek was the most poorly identified sample, with 61.5% accuracy (Supplementary Table 11). Talek was often mistaken for Upper Mara. Hippo samples were identified with 87.5% accuracy.

A mixing model (MixSIAR) using Bayesian inference and a Markov Chain Monte Carlo (MCMC) simulation was then utilized to provide the source proportions for each water sample filter[11]. An uninformative prior was utilized. Non-transformed means and standard deviations for the remaining elements that passed all prior statistical tests were input into the model for each of the 4 sources (Upper Mara, Middle Mara, Talek and Hippo). Non-transformed elemental concentrations were then input into the model for each sample. The model was run for 100,000 iterations with the first 50,000 discarded. We report means and 95% credibility intervals. Credibility intervals from a MCMC simulation with an uninformative prior distribution are similar to 95% confidence intervals[12]. The flux of suspended sediments derived from hippopotamus feces was calculated with the proportion estimates from the mixing model and the field measurements of TSS.

Supplementary Note 4.

Hippo pool water and feces collection

Hippo pool water (HPW) for the bottle experiment and stream experiment were collected from near the downstream portion of a hippo pool in the benthos of the pool (Amani, -1.29539, 35.205) and pumped into a black HDPE container using a peristaltic pump (6712C Compact Portable Sampler, Teledyne ISCO Lincoln, Nebraska), then sealed with an airtight lid. Samples were taken from Amani and preserved for analysis of H_2S (total dissolved sulfide), dissolved Fe(II), NH_4^+-N, SRP, NO_3^--N, TN, TP, DOC, dissolved gases (CO_2, CH_4, N_2O) and ions.

HPW for the hippo pool flushing experiment was transported to the reference pool by a truck with two 4000-L water tanks. 8000 L of HPW was pumped from near the bottom of an active hippo pool (Picnic Rock, -1.4338861, 35.3499556) into the water tanks. The truck transported the HPW 1 hour to the reference pool, which may have caused some aeration of the HPW, although it was still anoxic upon arrival. The HPW was then pumped directly into the reference pool. A total of 16,000 L was loaded into the reference pool over two days.

Samples for analysis were taken from the water tanks just prior to loading into the reference pool. The HPW was very high in NH_4^+-N, SRP, TN, TP, DOC, H_2S, CO_2, CH_4, cations, anions, and BOD5 (Supplementary Table 3).

Fresh hippopotamus feces were collected early in the morning from several bushes next to a hippo pool and then covered in a plastic container to prevent desiccation. HPW and hippopotamus feces were always collected within several hours of their use.

Supplementary References.

1. Kanga, E. M., Ogutu, J. O., Olff, H. & Santema, P. Population trend and distribution of the Vulnerable common hippopotamus Hippopotamus amphibius in the Mara Region of Kenya. *Oryx* **45**, 20-27, doi:10.1017/s0030605310000931 (2011).
2. Conley, D. J. *et al.* Hypoxia-Related Processes in the Baltic Sea. *Environ. Sci. Technol.* **43**, 3412-3420, doi:10.1021/es802762a (2009).
3. Hupfer, M. & Lewandowski, J. Oxygen Controls the Phosphorus Release from Lake Sediments – a Long-Lasting Paradigm in Limnology. *International Review of Hydrobiology* **93**, 415-432, doi:10.1002/iroh.200711054 (2008).
4. Kraal, P. *et al.* Decoupling between Water Column Oxygenation and Benthic Phosphate Dynamics in a Shallow Eutrophic Estuary. *Environ. Sci. Technol.* **47**, 3114-3121, doi:10.1021/es304868t (2013).
5. Liss, P. S. & Slater, P. G. Flux of Gases across the Air-Sea Interface. *Nature* **247**, 181-184, doi:10.1038/247181a0 (1974).
6. Benson, B. B. & Krause, D. The concentration and isotopic fractionation of gases dissolved in freshwater in equilibrium with the atmosphere. 1. Oxygen. *Limnology and Oceanography* **25**, 662-671, doi:10.4319/lo.1980.25.4.0662 (1980).
7. Dutton, C., Anisfeld, S. & Ernstberger, H. A novel sediment fingerprinting method using filtration: application to the Mara River, East Africa. *Journal of Soils and Sediments*, 1-16, doi:10.1007/s11368-013-0725-z (2013).
8. R: A language and environment for statistical computing v. 3.3.1 (R Foundation for Statistical Computing, Vienna, Austria, 2016).
9. Venables, W. N. & Ripley, B. D. *Modern applied statistics with S.* (Springer, 2002).
10. Weihs, C., Ligges, U., Luebke, K. & Raabe, N. in *Data Analysis and Decision Support* (eds D. Baier, R. Decker, & L. Schmidt-Thieme) 335-343 (Springer-Verlag, 2005).
11. MixSIAR GUI User Manual (2013).
12. McCarthy, M. A. *Bayesian Methods for Ecology*. (Cambridge University Press, 2007).
